# Hemorrhagic shock from breast blunt trauma

**DOI:** 10.1186/s12245-015-0083-2

**Published:** 2015-09-02

**Authors:** Bryan Madden, Mayura Phadtare, Zeina Ayoub, Ralphe Bou Chebl

**Affiliations:** Department of Emergency Medicine, Memorial Hermann Hospital, Houston, TX USA; Department of Emergency Medicine, American University of Beirut, Beirut, Lebanon

**Keywords:** Breast trauma, Hemorrhagic shock, Trauma

## Abstract

**Background:**

Seat belt use has been associated with decreased life-threatening thoracic injuries. However, there has been an increase in soft-tissue injuries such as breast trauma.

**Case report:**

We describe a case of a young healthy female who presented to a community hospital Emergency department without any trauma designation following a motor vehicle accident. The patient was found to have hemorrhagic shock from an intramammary hemorrhage and was treated with blood products and a temporizing external abdominal binder in preparation for a transfer to a level 1 center where she was successfully treated with angiographic embolization.

**Objectives:**

The objective of this study is to report on a case hemorrhagic shock from a breast hematoma as well as a review of the literature on previous seat belt associated breast trauma and its management in the emergency department.

**Conclusion:**

Seat belt associated breast trauma is uncommon in the emergency medicine literature. However, it can be associated with life threatening intramammary bleeding. Emergency physicians should be aware of these injuries and their proper management.

## Background

The use of seat belts has reduced the incidence of life-threatening chest trauma but has increased the incidence of soft-tissue and internal organ injuries [1, 2]. One of the resulting injuries is a blunt injury to the female breast. Blunt breast trauma literature is scarce in emergency medicine [3]. The aim of this paper is to report on a case of hemorrhagic shock resulting from a blunt breast trauma along with a review of the literature on the management of such an injury.

## Case presentation

A 54-year-old female with a history of hypertension and abdominal laparoscopy presented to a small community hospital without any trauma designation after a motor vehicle accident. The restrained patient was driving at high speed on wet roads when she lost control of her car and hit the front end of her vehicle on an embankment causing the car to roll over. The airbags did deploy. Patient was able to self extricate and was ambulatory on scene. The patient denied any loss of consciousness.

On arrival to the emergency department, the patient was anxious and complaining of right breast pain and right ankle pain. Her initial vitals were as follows: temperature 36.7 °C, heart rate 94 bpm, blood pressure 201/139 mmHg, respiratory rate of 20, and SaO2 100 % on room air. There were no signs of trauma on her head and neck area. There was a large contusion overlying the right breast with mild swelling when compared to the opposite breast. There was exquisite bilateral rib tenderness at multiple levels, and her right ankle was swollen and tender with intact pulses and sensation. The bedside focused assessment with sonography in trauma (FAST) exam was negative. She was given Fentanyl 100 mcg IV twice for pain control, normal saline 1 l IV, and taken to CT. CT head and cervical spine were unremarkable. CT angiography of her thorax demonstrated a 11.8 × 11.4 × 7.6 cm right breast hematoma with active extravasation. Upon return from CT, the patient was more diaphoretic, anxious, and hypotensive at 82/56 mmHg. She was immediately given a 2 l normal saline IV bolus, and massive transfusion protocol was activated. Because the patient’s level of pain was unchanged after Fentanyl, she was given Ketamine 150 mg IV. While sedated, her right breast was wrapped using an abdominal binder and elastic bandage as a temporizing compression measure. The patient was accepted for transfer to a level 1 trauma facility. At the time of transfer, she received two units of PRBCs and her vitals improved to a heart rate of 108 bpm and a blood pressure of 156/113 mmHg. Platelets and FFP were not ready at time of transfer. She was taken via ambulance because of weather conditions. Her labs were significant for an initial hemoglobin of 12.8 g/dL, a lactate of 2 mMol/L, drug screen positive for opiates, and negative ethanol level.

**Fig. 1:**
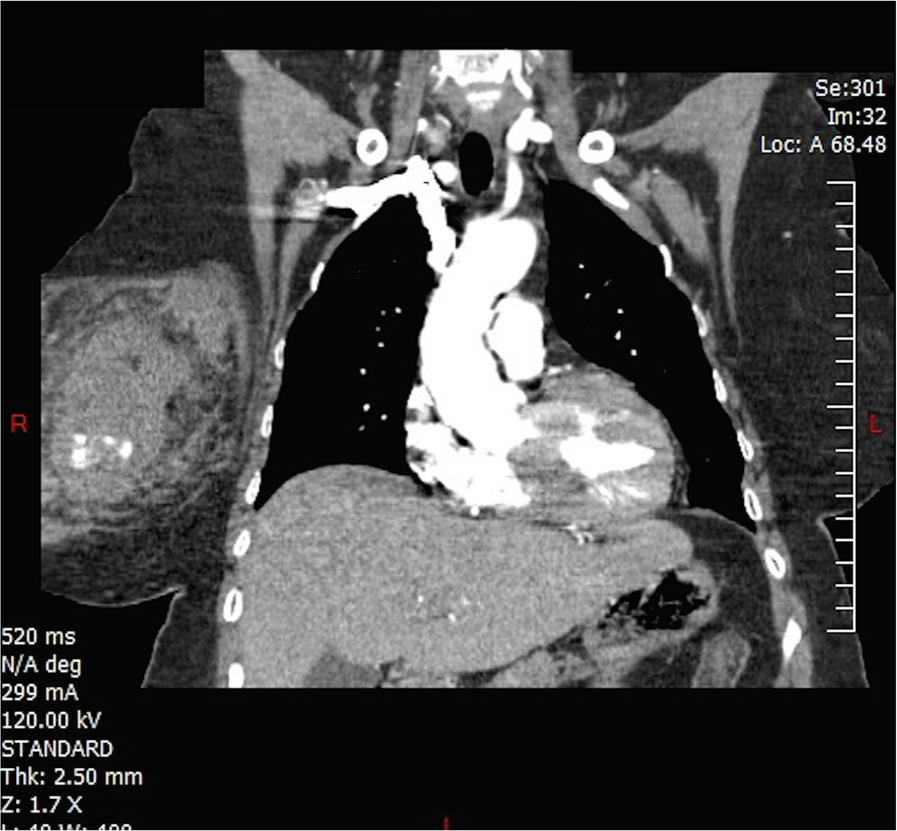
Coronal plane showing breast hematoma

**Fig. 2:**
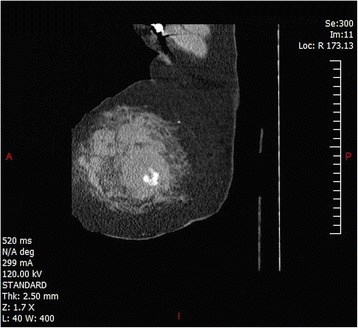
Breast hematoma in transverse plane

**Fig. 3:**
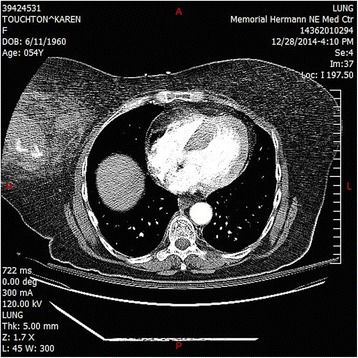
Breast hematoma sagittal plane with contrast extravasation

Upon arrival to the trauma center, her vitals were as follows: temperature of 36.1 °C, heart rate of 120 bpm, blood pressure of 130/80 mmHg, RR of 20, and a SaO2 of 100 % on nonrebreather. CT angiography of her chest was repeated along with a CT angiophraphy of her abdomen and pelvis. The CT once again showed the breast hematoma, (Figs. 1, 2 and 3) as well as left 4th–8th rib fractures, right 4th–7th rib fractures, and bilateral first rib fractures. Afterwards, she was taken to interventional radiology. Thoracic aortogram, internal mammary, and lateral thoracic branches arteriogram were negative for persistent extravasation. She received a total of three units of PRBCs as well as platelets and FFP during her hospitalization. Her hospital course was remarkable for her large opiate requirement. She was discharged 3 days after admission. Her Hemoglobin at discharge was 10.1 g/dL.

### Discussion

Breast injury is an uncommon form of blunt chest trauma. In a review of 5305 women with blunt chest trauma, only 108 (2 %) presented with breast trauma [4]. The mechanisms of breast trauma from a seat belt include both shear and crush injuries that result from the shoulder restraint. Majeski proposed a classification of breast trauma associated with seat belt injuries. From class 1 to 4, they ranged from mild bruising and tenderness to an avulsion of the breast from the chest wall with rupture of the blood vessels and active bleeding in to the chest [5]. The complete classification can be seen in Table 1. The most serious injuries to the breast are mammary duct avulsion and a vascular injury. The arterial supply to the breast comes from several sources, the internal mammary artery with perforators though the chest wall supplies the medial breast and the lateral thoracic branch of the axillary artery provides blood flow to the lateral breast. The majority of breast trauma patients had associated injuries. Of those, the most common were long bone extremity fractures (47 %), rib fractures (15 %), solid organ injury (11 %), and pneumothoraces/hemothoraces (10 %), all requiring chest tube placement [1]. Tam Song et al. reviewed all seat belt-related injuries and found 13 patients who presented to the emergency department at the time of the motor vehicle accident [2]. Of the immediate presentations, 4 patients had minor injuries such as lacerations and breast implant related injuries and were treated conservatively with outpatient plastic surgery follow-up. Nine patients required urgent attention and were found to have a rapidly expanding breast. Six of them deteriorated hemodynamically and were found to have arterial extravasation from the internal mammary artery, the lateral thoracic artery, and the accessory scapular branch of the axillary artery. Two pregnant patients had an enlarged breast due to accumulation of milk secondary to avulsed milk ducts. The last patient had an inflammatory air pocket in communication with an underlying pneumothorax, which resolved after chest tube placement. There is currently no established standard of management and treatment of blunt breast trauma. Patients should be assessed and treated like any major trauma patient following the ATLS guidelines. Sanders et al. proposed an algorithm based on their study [4]. Patients were divided as either having a simple or a complex breast trauma. Simple breast trauma patients defined as having an abrasion, a small laceration or pain over the affected breast were managed conservatively. Hemodynamically stable patients with complex breast trauma defined as a crush injury to the breast resulting in skin loss or an intramammary hematoma underwent a CT scan of their chest. Patients with no active arterial extravasation were monitored and treated symptomatically. Patients with a blush on CT were taken to interventional radiology for angiography and embolization [4]. Because this occurred at a community hospital without a trauma designation, both trauma services and timely interventional radiology were unavailable. The patient was successfully managed with blood product transfusion and a compressive band around the affected breast. The binder was used as a temporizing measure to provide external compression on the breast and to tamponade the bleed. The role of post traumatic mammography is controversial as it is generally unnecessary as long as clinical follow-up ensures resolution of any mass effect after recovery [1]. However, some authors advocate for a baseline mammogram at 3–6 months post injury with annual mammograms thereafter to ensure complete resolution of any masses and to rule out any post traumatic malignant breast malignancy [6]. Typical post traumatic mammographic findings involve fat necrosis in different stages of evolution that range from acute contusion to calcified oil cysts [2].

**Table 1:** Breast injury clasification

Grade 1	Mild crush injury consisting of bruising, ecchymosis, skin blistering, breast swelling, tenderness, friction burns over contact area.
Grade 2	Moderate crush injury consisting of intramammary hematoma, fat necrosis, skin avulsion or loss, skin laceration, skin ulcer
Grade 3	Severe crush injury consisting of subcutaneous partial or complete transection of the breast resulting in a permanent diagonal furrow across the breast corresponding to the line of the seat belt that cleaved the breast tissue into two parts
Grade 4	Avulsion breast injury consisting of subcutaneous avulsion of the breast from the chest wall with rupture of perforating branches of intercostal vessels, active bleeding into the breast and the space between the breast and chest wall caused by the traumatic shearing force

## Conclusions

This is an interesting case of hemorrhagic shock following a seat belt injury to the breast. The patient presented to a small community emergency department with normal vital signs. However, concern of rapid deterioration soon occurred after discovery of arterial extravasation of the breast and resultant hypotension. This is the first case report that shows the application of an abdominal binder on an actively bleeding intramammary hematoma, and in so, should be of relevance to emergency physicians.

## Consent

The patient has given her consent for the case report to be published.
